# Supplementary material for: Neural signatures of hyperdirect pathway activity in Parkinson’s disease
Source: Nat Commun. 2021 Aug 31;12:5185. doi: 10.1038/s41467-021-25366-0 (PMC8408177; doi:10.1038/s41467-021-25366-0)
Supplement: Supplementary file 1 — Supplementary Information [file 41467_2021_25366_MOESM1_ESM.pdf]

Supplementary Information

Supplementary Methods

Surgical Procedure

At UCL a Medtronic (Medtronic Neurological Division, Minneapolis, MN) model 3389 electrode was implanted in all but one subject (GPi 1), who was implanted with the Medtronic model 3387 electrode. At Shanghai in contrast a Medtronic model 3387 electrode was implanted in all patients. Each electrode comprised four platinum-iridium contacts (model 3389 – diameter 1.27 mm, length 1.5 mm, centre-centre separation 2 mm; model 3387 – diameter 1.27 mm, length 1.5 mm, centre-centre separation 3 mm) that are numbered from 0 (lowermost) to 3 (uppermost). The surgical targets investigated here were the dorsal motor region of the STN and the posterior third of the ventral pallidum in the two cohorts. Further details of the operative procedure at UCL and Shanghai can be found in related works [1–4].

At UCL, the locations of the electrodes were confirmed following implantation with immediate postoperative fast spin-echo T2-weighted magnetic resonance imaging (MRI 1.5 T) with a Leksell frame still in situ. In Shanghai immediate postoperative CT co-registered to a preoperative 3T MRI was used to confirm electrode localisation [3]. At both centres stainless steel electrode extension cables were externalized through the scalp to enable recordings prior to connection to a subcutaneous DBS pacemaker, implanted in a second operative procedure seven days later. Recordings were performed between days 3–6 after electrode implantation.

Simultaneous magnetoencephalography and local field potential recordings

For the UCL cohort MEG recordings were performed using a CTF 275-channel MEG system (CTF/VSM MedTech). MEG data were sampled at 2400 Hz and stored to disk for subsequent offline analyses. LFP activity recorded from DBS electrodes was collected at the same time as MEG using a battery-powered and mains optically isolated BrainAmp system (Brain Products). Three bipolar channels (0-1, 1-2, 2-3) were recorded from each electrode and were high-pass filtered at 1 Hz in the hardware to avoid amplifier saturation due to large DC offsets. In Shanghai MEG was recorded with a 306-channel MEG scanner (Elekta Oy, Helsinki, Finland). The integrated EEG system was used to record the three bipolar channels from each electrode as above. Raw data were sampled at 1000 Hz and band pass filtered in 0.03–330 Hz range and digitized at 1,000 Hz.

Recordings were performed either in the ON or in the OFF medicated state as detailed in the Methods section of the main manuscript. The patient was requested to stay still during recordings and to keep their eyes open whilst focussing on a fixation cross that appeared on a screen in front of them. Rest recordings had a duration of 3 minutes and merged MEG-LFP data were epoched into trials of 3 second duration which were subsequently averaged after the computation of auto- and cross- spectral measures. A neurologist was present during recordings in order to ensure patient safety.

Generation of volumetric coherence images using beamforming

Beamforming relies on a linear projection of sensor data using a spatial filter that is computed from the lead-field of a location of interest and either the data covariance or the cross-spectral density matrix [5,6]. Lead-fields were computed using a single shell head model [7]. The model was generated in SPM12 based on the patient's preoperative structural MRI and fiducial-based co-registration was performed.

The source space was defined as a 5 mm spaced grid in MNI (Montreal Neurological Institute) space bounded by the inner skull surface. The resulting values of coherence – between each grid point and the subcortical LFP - were linearly interpolated to produce 3D volumetric images with 2 mm resolution for visualisation. Coherence images were smoothed with an 8 mm isotropic Gaussian kernel to ensure conformance to the assumptions of random field theory prior to statistical analysis in SPM.

Estimation of directionality

The effective directionality of coupling between the cortex and the STN/GPi LFP was computed with a non-parametric variant of spectral Granger causality [8,9]. To determine the significance of directionality estimates, we compared the Granger estimate of original data to that of surrogate time-reversed data using a *t*-test [10] with a significance threshold set to 0.05. Taking the example of two signals A and B, with A Granger causing B, the Granger causality from A to B should be higher for the original than for the time-reversed data, giving rise to a positive difference. In contrast, the estimate of causality from B to A should be increased by time reversal thereby giving a negative difference. In the event of there being statistically significant coupling in one particular direction (e.g. cortex to sub-cortex), time delays were computed by regressing the unwrapped angle of the cross-spectrum between the two component signals against frequency using the regress function in MATLAB with a significance threshold for slope estimation of 0.05 [11]. Time delays were statistically compared for the STN and GPi with covariates to account for both subject-specific dependencies in the recordings from the two hemispheres (between subjects), and for potential differences between the recordings from the right and left sides in individual subjects (within subjects). Covariates representing each patient's preoperative levodopa

equivalent dose, UPDRS Part III motor score on medication and MMSE were also introduced in order to account for phenotypic differences between the two groups.

Preoperative diffusion MRI acquisition and pre-processing

For eight patients studied (STN UCL patients 7-14; see Supplementary Table 1) preoperative diffusion MRI data were acquired on a 3T Siemens Magnetom Trio TIM Syngo MR B17 using a padded 32-channel receive head coil to reduce discomfort and head motion. Further details on acquisition and pre-processing maybe found in[12]. Siemens' 511E-Advanced Echo Planar Imaging Diffusion WIP was used. In-plane acceleration was used (GRAPPA factor of 2) with partial Fourier 6/8. In-plane resolution was 1.5×1.5 mm^2 (Field of view 219×219 mm^2, TR = 12,200 ms, TE = 99.6 ms) and 85 slices were acquired with a 1.5 mm thickness. Diffusion-weighting with $b = 1500$ s/mm^2 was applied along 128-directions uniformly distributed on the sphere and seven $b = 0$ s/mm^2 volumes were acquired. To correct for distortions all acquisitions were repeated with reversed phase encoding direction (left to right and right to left phase encode) giving a total of 270 volumes acquired ($[128 + 7] \times 2$). The total acquisition time for the dMRI sequences was 62 min.

The diffusion data were acquired with reversed phase-encode blips (left-to-right and right-to-left), resulting in pairs of images with distortion going in opposite directions. From these pairs, the susceptibility induced off-resonance field was estimated using a method implemented in the FSL software package [13,14] and the two images were combined into a single corrected one using Topup as implemented in FSL v5.0. The output from Topup was then fed into Eddy (FSL v5.0) for correction of eddy current distortions and subject movement [15].

Computational modelling of high and low beta band oscillatory synchrony

The model developed here rests on the premise that the change in the average firing rate of a population *i*, $F_i'(t)$ is governed by the population time constant, τ the current firing rate, $F_i(t)$ and a sigmoidal function, S of the sum of all delayed synaptic inputs, $I_{ji}(t-T_{ji})$ from sources *j* to population *i* multiplied by their respective connection strengths, w_{ji} [16]:

(1) $$\tau \frac{dF_i}{dt} = \tau F_i'(t) = S_i\left(\sum_j w_{ji} I_{ji}(t-T_{ji})\right) - F_i(t)$$

The sigmoid function, S_i in (1) represents the activation function of population *i* in response to synaptic inputs and is parameterised by the maximal firing rate of the population (M_i) and its firing rate in the absence of inputs (B_i) as indicated for each population in (3).

Additional features which define the sigmoid function are the firing rate in response to zero input and the maximal slope. We modelled the dynamics of five neuronal populations within the cortico-basal-ganglia circuit: 1) An excitatory cortical population, E ,2) an inhibitory cortical population, I, 3) the STN, 4) the GPe and 5) the GPi. **Supplementary Figure 6A** provides an illustration of modelled populations and their connections. In keeping with the work of Pavlides et al., 2015 cortical activity was modelled with an excitatory and inhibitory population, with the former providing inputs to the basal ganglia. Additionally in the present model we included: 1) an auto-inhibitory self-connection in the inhibitory cortical neural population which has been considered in previous accounts of cortical Jansen-Rit type neural mass models [17], 2) an explicit model of the dynamics of the GPi based on its anatomical connectivity [18,19] as our primary motive was to compare the activity and connectivity profile of the STN and GPi and 3) an additive signal dependent stochastic noise term so that the differential equations describing the evolution of firing rates for each population were stochastic delay differential equations (SDDEs) [20,21]. The addition of signal dependent noise

serves to ensure that the spike rate variance correlates with the mean firing rate at each integration step, ΔT (see for example Dayan and Abbott, 2001 for a discussion of the Fano factor).

Rather than explicitly modelling the firing dynamics of the striatum we considered inhibitory striatal inputs, *S* to the GPi and GPe to be fixed in keeping with [22]. Similarly the excitatory cortical population, *E* receives a constant component of intrinsic and extrinsic excitatory inputs, *C*. We also consider that the net effect of excitation of the GPi-thalamo-cortical loop has an inhibitory effect on excitatory cortical population activity. The equations governing the dynamics of the cortico-basal-ganglia circuit in our model are as follows, where $W(t)$ represents the standard Weiner process:

(2)

$$dE(t) = \frac{F_E(-w_{GiE}GPi(t-T_{GiE}) - w_{IE}I(t-T_{IE}) + C) - E(t)}{\tau_E} dt + E(t)dW_E(t)$$

$$dI(t) = \frac{F_I(w_{EI}E(t-T_{EI}) - w_{II}I(t-T_{II})) - I(t)}{\tau_I} dt + I(t)dW_I(t)$$

$$dS(t) = \frac{F_S(w_{ES}E(t-T_{ES}) - w_{GeS}GPe(t-T_{GeS})) - S(t)}{\tau_S} dt + S(t)dW_S(t)$$

$$dGPe(t) = \frac{F_{GPe}(w_{SGe}S(t-T_{SGe}) - w_{GeGe}GPe(t-T_{GeGe}) - Str) - GPe(t)}{\tau_{GPe}} dt + GPe(t)dW_{GPe}(t)$$

$$dGPi(t) = \frac{F_{GPi}(-w_{GeGi}GPe(t-T_{GeGi}) + w_{SGi}S(t-T_{SGi}) - w_{GiGi}GPi(t-T_{GiGi}) - Str) - GPi(t)}{\tau_{GPi}} + GPi(t)dW_{GPi}(t)$$

The sigmoid functions governing the activation function of each population are as follows:

(3)

$$F_E(x) = \frac{M_E}{1 + \left(\frac{M_E - B_E}{B_E}\right)\exp\left(\frac{-4x}{M_E}\right)}$$

$$F_I(x) = \frac{M_I}{1 + \left(\frac{M_I - B_I}{B_I}\right)\exp\left(\frac{-4x}{M_I}\right)}$$

$$F_S(x) = \frac{M_S}{1 + \left(\frac{M_S - B_S}{B_S}\right)\exp\left(\frac{-4x}{M_S}\right)}$$

$$F_{GPe}(x) = \frac{M_{GPe}}{1 + \left(\frac{M_{GPe} - B_{GPe}}{B_{GPe}}\right)\exp\left(\frac{-4x}{M_{GPe}}\right)}$$

$$F_{GPi}(x) = \frac{M_{GPi}}{1 + \left(\frac{M_{GPi} - B_{GPi}}{B_{GPi}}\right)\exp\left(\frac{-4x}{M_{GPi}}\right)}$$

The resulting SDDEs were integrated over a time period of 4 seconds using a Euler-Maruyama integration scheme [20] with a step size, ΔT of 10^{-4} seconds where the additive noise term is given by: $W_i(t+\Delta T) - W_i(t) \sim N\left(0, \sqrt{\Delta T}\right)$.

Simulations with noise were performed to ensure physiological values of coherence (**Figure 7**). However, we also investigate the behaviour of this system without noise by integrating the above equations in the absence of the noise term using the built-in MATLAB integrator, DDE23 (**Figure 6 and Supplementary Figure 6**). In contrast to [22] who focus purely on the generation of broad band beta oscillations our aim was to capture mechanisms underlying the generation of low and high beta band activities that could explain both our data and those of other studies. Where possible, parameters of our model were selected from those previously detailed in [22] (see **Supplementary Table 2** for further details of parameters used).

Supplementary Results

Simultaneous visualisation of cortico-subcortical fibre tracts and MEG networks

Supplementary Figure 3 displays the intersection of tractography derived structural connectivity and MEG derived functional connectivity. The magenta streamlines indicate tracts passing between the broadband beta network (shown in yellow) and the STN (left panel) and GPi (right panel). The green streamlines indicate tracts passing between the high beta network (shown in red) and the STN and GPi. Since voxels comprising the high beta network were contained within those comprising the broadband beta network, the former were subtracted from the latter to highlight the difference in associated tracts. The colour bars indicate the number of repetitions of each fibre across subjects.

Comparison of cortico-STN/GPi tract densities within MEG derived beta band networks

Tract densities were computed for the ipsilateral and contralateral high beta and broadband beta networks, separately for STN and GPi contacts (**Figure 6C** and main **Results** section). We explored this phenomenon statistically by constructing a 2 x 2 x 2 factorial ANOVA with factors network (high beta vs. broadband beta), laterality (ipsilateral vs. contralateral) and electrode location (STN vs. GPi).

For the UCL cohort significant main effects were observed for all three factors (high beta vs. low beta: $F(1,223)$ =63.46.16, $P < 10^{-4}$; ipsilateral vs. contralateral: $F(1,223)$ =155.0, $P < 10^{-4}$; STN vs. GPi: $F(1,223)$ =168.29, $P < 10^{-4}$). Additionally, all pairwise interactions and the three way interaction were significant, (interaction of electrode location and laterality: $F(1,223)$ = 56.77.46, $P < 10^{-4}$; interaction of electrode location and network: $F(1,223)$ = 32.83, $P < 10^{-4}$; interaction of network and laterality $F(1,223)$ = 59.12, $P < 10^{-4}$; interaction of network, laterality and electrode location $F(1,223)$ = 32.59, $P < 10^{-4}$), highlighting that the difference in tract density between the high beta and broadband beta networks was greater for

the ipsilateral rather than the contralateral STN, and that this effect was less marked for the GPi.

In contrast for the Shanghai cohort significant main effects were observed for network (high beta vs. low beta: $F(1,247) = 116.38$, $P< 10^{-4}$) and laterality (ipsilateral vs. contralateral: $F(1,247) = 249.03$, $P< 10^{-4}$) but not for electrode location (STN vs. GPi: $F(1,247) = 2.09$, $P= 0.15$). The interactions between electrode location and network ($F(1,247) = 2.41$, $P =0.12$) and three way interaction between network, laterality and electrode position were not significant ($F(1,247) = 2.41$, $P =0.11$). There were however significant interactions between electrode location and laterality ($F(1,247) = 4.19$, $P =0.04$) and between laterality and network ($F(1,247) = 107.41$, $P < 10^{-4}$) highlighting that tract densities were greater for ipsilateral rather than contralateral connections and that this effect was greater for: 1) the STN rather than the GPi and 2) for the high beta network rather than the overall beta network. The results from the Shanghai cohort are therefore slightly different, but reasonably consistent to those from the UCL cohort (see **Discussion** for further comment).

Finally for the separate cohort of UCL patients with both individual structural connectomes and combined MEG-LFP recordings (UCL STN patients 7-14; see **Supplementary Table 1**), we compared tract densities using a 2x2 ANOVA with factors network (high beta vs. broadband beta) and laterality (ipsilateral vs. contralateral). As per the PPMI connectomes we observed significant main effect of both laterality ($F(1,165) = 40.52$, $P < 10^{-4}$) and network ($F(1,165) = 26.10$, $P < 10^{-4}$) such that tract densities were greater for the high beta network and for the ipsilateral side. Additionally there was an interaction between the two factors such that the difference in tract densities for the ipsilateral and contralateral sides was greater for the high beta network than for the overall beta network ($F(1,165) = 40.52$, $P < 10^{-4}$).

Computational model: cortically generated high beta frequency activity can induce the generation of lower frequency beta rhythms in the subcortex.

Our model was set up to account for the observation that high beta activity generated in the cortex propagates subcortically to the STN (see left image in **Supplementary Figure 4A** for the structure of the model). This is in keeping both with our present analysis of cortico-STN directionality in the high beta frequency range and with the results of other reports [11,23,24]. The middle and rightmost images in **Supplementary Figure 4A** display the cortical generation of high beta frequency oscillations. In this simulation we consider only the cortical excitatory (E) and inhibitory (I) populations and therefore the top-down and bottom-up connections (W_{ES} and W_{GiE}) to and from the basal ganglia are set to zero. The middle image in (A) details the effect of changing the coupling parameters, $W_{IE/EI}$ on the peak frequency generated by the cortical populations. The right most image in (A) shows simulated firing rates and corresponding spectra for the cortical populations with the coupling parameter, $W_{IE/EI}$ set to a value of 4. In this instance both the E and I populations produce oscillatory activity with a frequency of approximately 27 Hz. The remaining parameters in this simulation are as per **Supplementary Table 2**.

Secondly, in our model low beta frequencies are generated by delayed interactions in the reciprocal loop between the STN and the GPe. It has previously been shown that this loop is capable of producing oscillations under certain conditions when the system transitions from a stable to an unstable fixed point through a Hopf bifurcation [22,25]. **Supplementary Figure 4B** shows a simulation similar to that performed in **Supplementary Figure 4A**, but this time with the introduction of top-down (W_{ES}) and bottom-up connections (W_{GiE}) to and from the basal ganglia. We use fixed values of W_{ES} and $W_{EI/IE}$ but vary the strength of the net

inhibitory loop between the GPi, thalamus and cortex, W_{GiE}. In each of the three images in **Supplementary Figure 4B**, the top subplot displays the integrated time series, whilst the middle subplot shows the resulting power spectra and the bottom subplot a phase portrait of STN and GPe firing rates after the system reaches the vicinity of a periodic orbit. In these phase portraits activity of GPe within a low beta oscillation cycle is plotted against the activity in STN, and different points on the curve correspond to different time points within a cycle. The phase portraits are colour coded depending on the input excitatory cortical population firing rate, thus the two yellow segments of the curve correspond to peaks of cortical high beta oscillation. At low values of W_{GiE} the time series of STN, GPi and GPe comprise high and low beta frequency components (best seen in the left image in **Supplementary Figure 4B**) that are observed in the time series, the spectra and the profile of the STN/GPe phase portraits. In summary, the model including two generators of oscillation in cortex and STN-GPe circuit qualitatively reproduced the two peaks in beta activity seen in **Figures 2, 3 and 4**.

Comment on bursting behaviours

Recent work has focussed on the importance of transient rather than sustained episodes of synchrony being important in both pathological and physiological states [26–28]. Although the model presented here does not explicitly focus on the generation of bursting behaviours, it is easy to see how bursts may arise. Endogenous fluctuations (triggered by noise) of the inputs to the reciprocal STN-GPe loop when it is operating close to its bifurcation point may trigger transient oscillatory behaviours (see the left image in **Figure 8B**). This model therefore makes the testable prediction that transient bursts of cortical high beta activity can trigger the generation of lower beta frequency bursts within the STN. The genesis of bursting activity within similar models is likely to be a focus of future work.

Supplementary Figures

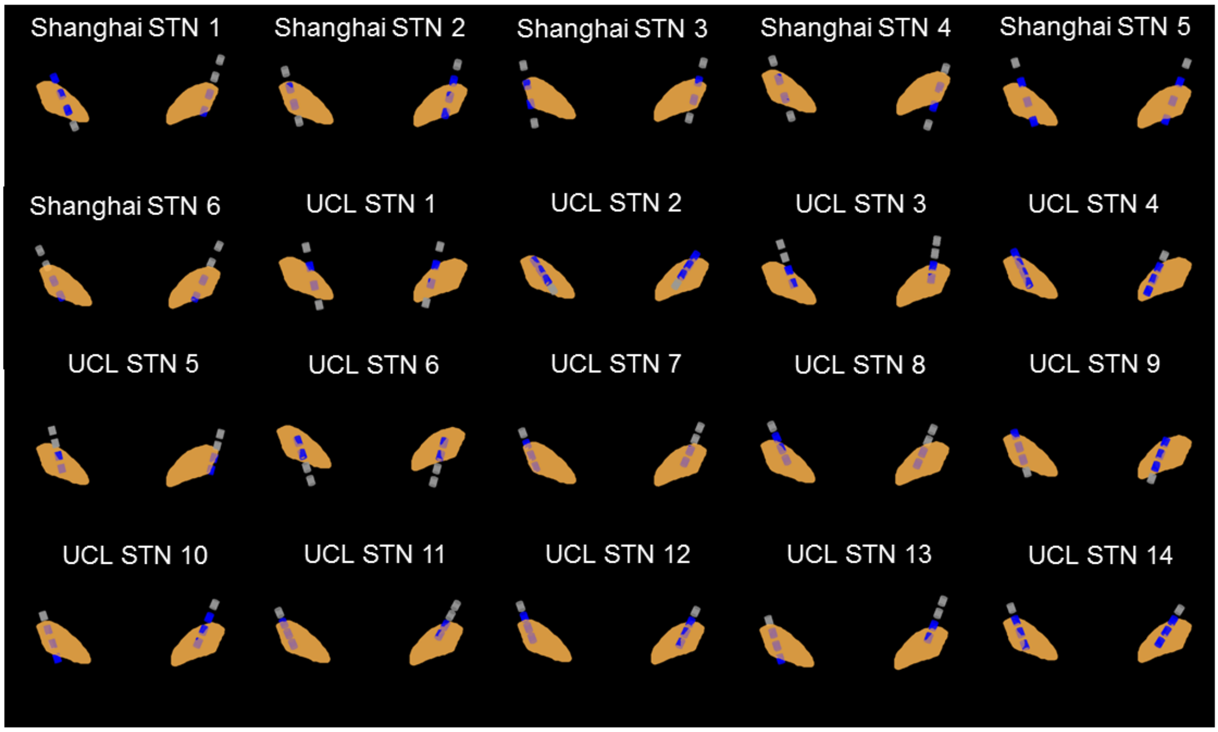

Supplementary Fig 1. Individual STN contact localisations

All STN contacts are visualised in MNI template space in the coronal plane (viewed from posterior to anterior), individually for each of the twenty STN DBS patients in this report (see **Supplementary Table 1** for further details). Templates of the STN are coloured orange, whilst electrode contacts traversing the STN are coloured in blue. Contacts not traversing the STN are coloured grey. Only data from adjacent contact pairs, where at least one contact traversed the STN was used for subsequent analysis.

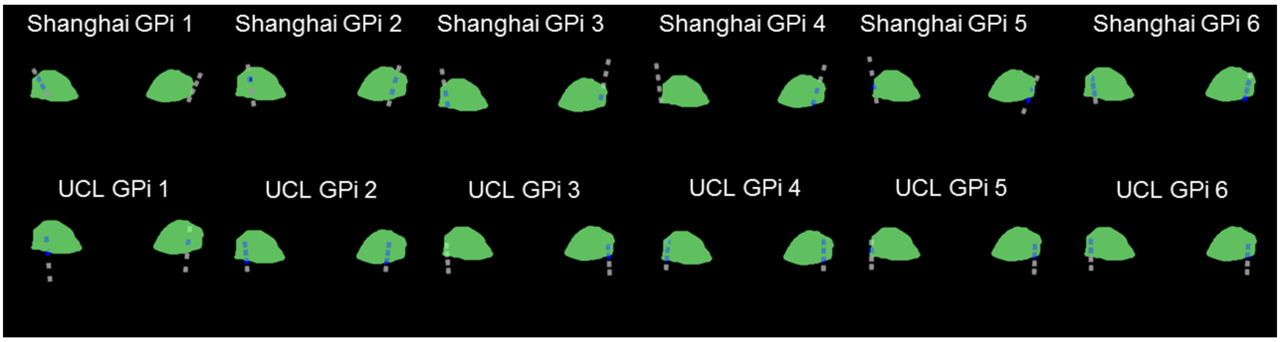

Supplementary Fig 2. Individual GPi contact localisations

All GPi contacts are visualised in MNI template space in the coronal plane (viewed from posterior to anterior), individually for each of the twelve GPi DBS patients studied in this report. Templates of the GPi are coloured green, whilst electrode contacts traversing the GPi are coloured in blue. Contacts not traversing the GPi are coloured grey. Only data from adjacent contact pairs, where at least one contact traversed the GPi was used for subsequent analysis.

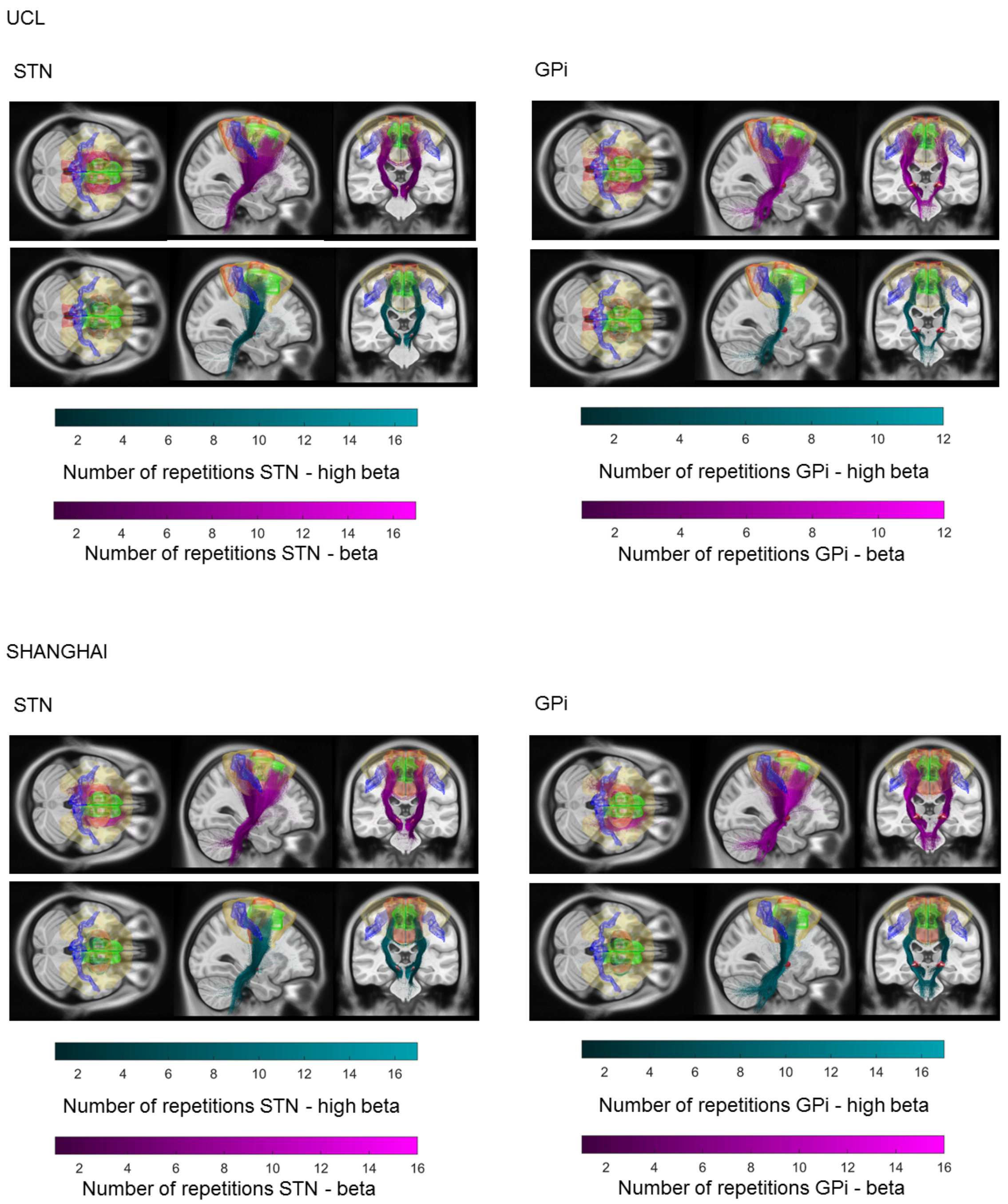

Supplementary Fig 3. Intersection of group level MEG derived cortico-STN/cortico-GPi networks and PPMI derived tractography streamlines for the UCL (upper panel) and Shanghai (lower panel) cohorts. Cortico-STN and cortico-GPi networks derived from concurrent MEG and LFP recordings are displayed together with fibre streamlines on a T1-weighted MRI scan. The left panel depicts fibres passing to STN contacts, whilst the right panel shows fibres passing to GPi contacts. In each case, fibres were selected to originate in

either: 1) cortical regions where there was a significant main effect of band for the STN (termed the high beta network which is shown in red with fibres originating from it coloured green) or 2) cortical regions that couple to the STN and GPi across the entire beta frequency range (termed the beta network which is shown in yellow with fibres originating from it coloured in magenta). Fibres are colour coded depending on the number of times they were repeatedly chosen (see colour bar). Cortical regions shown in blue and green, indicate boundaries of the primary motor cortex and SMA respectively.

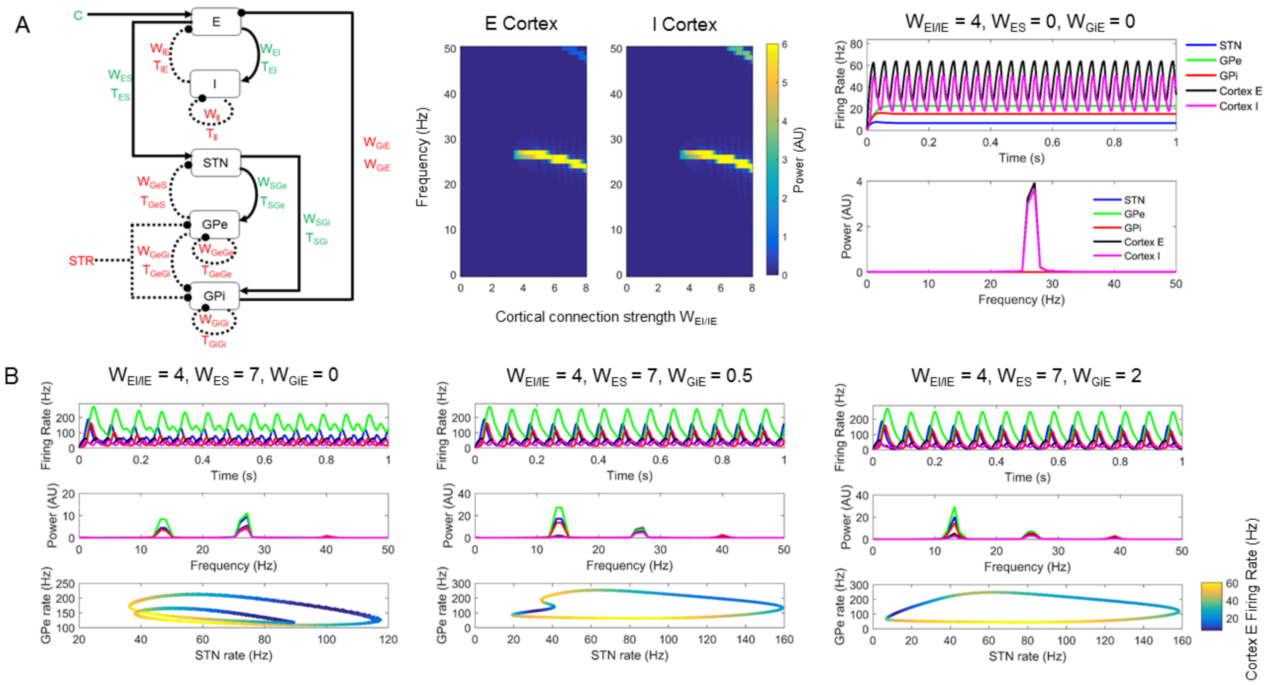

Supplementary Fig 4. Computational model of the generation of cortical high beta frequency activity which can in turn trigger resonance at lower beta frequencies within the STN-GPe loop. (A): the left image displays the connectivity of the cortico-basal-ganglia circuit in the computational model. The computational model developed includes the major connections of the basal ganglia. Excitatory connections are depicted by filled black lines with arrows, whilst inhibitory connections are indicated by dashed black lines with circles. E = cortical excitatory population, I = cortical inhibitory population, S = STN, Ge = GPe and Gi = GPi. W_{ES} and T_{ES} indicate the strength and delay of synaptic connections between the excitatory cortical population and the STN. Inhibitory inputs, connections and delays are coloured red, whilst excitatory inputs, delays and connections are coloured green. Inhibitory striatal (Str) inputs to GPe and GPi are considered to be fixed in our model. An input, C representing a constant component of intrinsic and extrinsic excitatory inputs is provided to the excitatory cortical population, E. The inhibitory cortical population, I, the GPe and the GPi have self-inhibitory connections with delays. Differential equations governing the

dynamics of studied nodes are detailed in equation 1. The middle and right images of (A) reveal the behaviours of the excitatory cortical population(E), the inhibitory cortical population (I), the STN, the GPe and the GPi in the absence of top-down and bottom-up connections to and from the basal ganglia. This is equivalent to setting, W_{ES} and W_{GiE} equal to 0. Simulations are performed in the absence of additive noise. The middle image in (A) shows the effect of changing the coupling parameters, $W_{IE/EI}$ on the peak frequency generated by the cortical populations. The right image in (A) shows simulated firing rates and corresponding spectra for the cortical populations with the coupling parameter, $W_{IE/EI}$ set to a value of 4. In this simulation, the remaining parameters were set to those detailed in **Supplementary Table 2**. With W_{EI} and W_{IE} set to 4 the cortical population is capable of generating oscillatory activity in the high beta frequency range (approximately 27 Hz). In Panel (B) top-down (W_{ES}) and bottom-up connections (W_{GiE}) to and from the basal ganglia are introduced. We use fixed values of W_{ES} and $W_{EI/IE}$ but vary the strength of the net inhibitory loop between the GPi, thalamus and cortex (W_{GiE}). For each of the three images in panel B, the top subplot shows the integrated time series (firing rates) for the excitatory cortical population (E), the inhibitory cortical population (I), the STN, the GPe and the GPi. The middle subplot shows power spectra of the integrated time series and the bottom subplot shows a phase portrait of STN and GPe activities colour coded by the firing rate of the excitatory cortical population, E, which provides inputs to the STN. At low values of W_{GiE} the time series of STN, GPi and GPe comprise high and low beta frequency components which are also reflected in the periodic orbits of the phase portraits.

Supplementary Tables

Case	State during recording	Age/Sex/ Handedness	PD Duration (Years)	Preoperative Medication (LDE mg)	Preoperative UPDRS III (On/Off medication) /Symptom dominant side	Cognitive Score (MMSE)
STN UCL 1	ON/OFF	51/M/R	8	650	(21/49) L	29
STN UCL 2	ON/OFF	54/M/R	8	2150	(9/38) R	29
STN UCL 3	ON/OFF	58/M/L	11	1320	(25/43) L	30
STN UCL 4	ON/OFF	54/M/R	15	1150	(19/53) R	28
STN UCL 5	ON/OFF	55/M/R	24	1320	(5/19) L	29
STN UCL 6	ON/OFF	52/M/R	13	1080	(10/35) L	28
**Mean**		**54**	**13.2**	**1278**	**14.8/39.5**	**28.8**
STN UCL 7	OFF	43/M/R	9	1400	(--/63) R	29
STN UCL 8	OFF	61/M/L	8	400	(--/56) L	29
STN UCL 9	OFF	62/M/R	14	1640	(--/39) L	30
STN UCL 10	OFF	60/M/R	8	456	(--/46) R	30
STN UCL 11	OFF	41/M/R	6	1020	(--/50) L	29
STN UCL 12	OFF	58/M/R	12	1200	(--/38) R	29
STN UCL 13	OFF	59/M/R	13	1900	(--/48) L	29
STN UCL 14	OFF	67/F/R	15	1120	(--/32) L	29
**Mean**		**56.4**	**10.6**	**1142**	**--/46.5**	**29.3**
GPi 1 UCL	ON	61/M/R	14	500	(27/--) R	25
GPi 2 UCL	ON	75/M/R	11	670	(26/--) R	24
GPi 3 UCL	ON	73/M/L	15	380	(42/--) L	25
GPi 4 UCL	ON	65/M/R	11	923	(33/--) L	25
GPi 5 UCL	ON	46/M/R	10	575	(37/--) R	22
GPi 6 UCL	ON	71/M/R	15	833	(16/--) L	21
**Mean**		**65.2**	**12.7**	**647**	**30.2/--**	**23.7**
**UCL STN 1-6 vs. GPi 1-6**		**T_{10} =2.48 p=0.03**	**T_{10} =0.19 p=0.85**	**T_{10} =2.89 p=0.02**	**T_{10} =3.10 p=0.01**	**T_{10} =6.64 p<0.01**
STN SHA 1	ON	72/M/R	5	750	(31/--)R	27
STN SHA 2	ON	50/F/L	4	1000	(22/--)L	27
STN SHA 3	ON	63/M/R	10	750	(42/--)L	28
STN SHA 4	ON	65/F/R	12	400	(25/--)R	27
STN SHA 5	ON	68/M/R	12	1100	(31/--)R	27
STN SHA 6	ON	64/M/R	14	816	(11/--)L	28
**Mean**		**63.7**	**9.5**	**803**	**27**	**27.3**
GPi SHA 1	ON	48/F/L	5	600	(35/--)R	28
GPi SHA 2	ON	54/F/R	7	550	(26/--)R	25
GPi SHA 3	ON	68/M/R	20	975	(34/--)L	28
GPi SHA 4	ON	58/M/R	4	750	(41/--)L	27
GPi SHA 5	ON	43/M/R	17	400	(23/--)R	28
GPi SHA 6	ON	57/F/R	12	800	(15/--)L	27
**Mean**		**54.7**	**10.8**	**679**	**29**	**27.2**
**SHA STN 1-6 vs. GPi 1-6**		**T_{10} =1.93 p=0.08**	**T_{10} =0.42 p=0.68**	**T_{10} =0.95 p=0.36**	**T_{10} =0.35 p=0.73**	**T_{10} =0.32 p=0.76**

Supplementary Table 1. Clinical characteristics of patients. Data from STN UCL patients 1-6 was compared to data from GPi UCL patients 1-6. Similarly, STN Shanghai (SHA) patients 1-6 were compared with GPi Shanghai patients 1-6. Phenotypical features of the STN and GPi patient groups were compared using an unpaired T-test – the t statistic and p value (two-tailed) for each comparison are shown in the table. Data from STN UCL patients 7-14 was used in the correlation of individual subject tractography datasets with MEG and STN LFP data. LDE - levodopa dose equivalent. The total pre-operative UPDRS III [29]motor score is presented with medication state depending on whether recordings were performed on, off or both on and off medication. Cognitive function was assessed with the the Mini-Mental State Examination, MMSE [30].

Parameter	Value	Comment/Reference
**Fixed inputs**		
C	172.18	[22]
Str	8.46	[22]
**Synaptic connection weight (AU)**		
W_{IE}/W_{IE}	4	[22]
W_{ES}	7	[22]
W_{II}	3	We reasoned that strength of auto-inhibition might be less than that of inhibition of excitatory population as per [17] who study cortical columns in a Jansen-Rit model
W_{GeS}	1.3	[22]
W_{SGe}	4.87	[22]
W_{GeGe}	0.53	[22]
W_{SGi}	4	We expect that this would be similar to W_{SGe} based on tractography and cellular studies of STN efferents to GPi/GPe [31–33]
W_{GeGi}	1	We reasoned that this would be stronger than W_{GeGe} based on higher density of synapses from GPe neurones to GPi neurones than to GPe neurones [32]
W_{GiGi}	0.53	We account for the possibility that GPi neurones may inhibit their own activation via recurrent connections [32,34]
W_{GiE}	0.5	We explored a number of values for this parameter and conclude that the net inhibitory effect of activation of the GPi-thalamo-cortical loop is small in keeping with previous studies [35–37]
**Delays (ms)**		
T_{IE}/T_{IE}	5	Mean of range previously reported [22]
T_{II}	4	We reasoned that auto-inhibitory delays between cells of the same population are fixed. We therefore used constant values for T_{II}, T_{GiGi} &T_{GeGe}
T_{ES}	5.5	[22]
T_{GeS}	6	[22]
T_{SGe}	6	[22]
T_{GeGe}	4	[22]
T_{SGi}	6	Similar to T_{GeS} based on evoked responses to STN stimulation [38]

T_{GeGi}	6	Similar to T_{SGe} based on latency of inhibitory response of STN→GPe→GPi following single pulse stimulation of STN [38]
T_{GiGi}	4	We reasoned that auto-inhibitory delays between cells of the same population are fixed. We therefore used constant values for T_{II}, T_{GiGi} &T_{GeGe}
T_{GiE}	20	Within the range delays previously reported [39]
**Time constants (ms)**		
τ_E	11	[22]
τ_I	11	[22]
τ_S	12.8	[22]
τ_{GPe}	20	[22]
τ_{GPi}	14	[19]
**Sigmoid function firing parameters (spikes/s)**		
M_E/B_E	75/17.85	[22]
M_I/B_I	205/9.87	[22]
M_s/B_s	300/10	[22]
M_{GPe}/B_{GPe}	400/10	[22]
M_{GPi}/B_{GPi}	400/18	Taken from in vitro recordings and responses to stimulation [19,38]

Supplementary Table 2. Parameter values used for generating simulated data from the computational model. The majority of parameter values are derived from [22]. Where we have introduced additional parameters, we provide a comment or reference justifying selection of the particular value.

Supplementary References

1. Foltynie, T. & Hariz, M. I. Surgical management of Parkinson's disease. *Expert Rev. Neurother.* **10**, 903–14 (2010).
2. Foltynie, T. *et al.* MRI-guided STN DBS in Parkinson's disease without microelectrode recording: efficacy and safety. *J. Neurol. Neurosurg. Psychiatry* **82**, 358–63 (2011).
3. Zhan, S. *et al.* Bilateral deep brain stimulation of the subthalamic nucleus in primary Meige syndrome. *J. Neurosurg.* **128**, 897–902 (2018).
4. Sun, B., Chen, S., Zhan, S., Le, W. & Krahl, S. E. Subthalamic nucleus stimulation for primary dystonia and tardive dystonia. *Acta Neurochirurgica, Supplementum* **97**, 207–214 (2007).
5. Van Veen, B. D., van Drongelen, W., Yuchtman, M. & Suzuki, A. Localization of brain electrical activity via linearly constrained minimum variance spatial filtering. *IEEE Trans. Biomed. Eng.* **44**, 867–80 (1997).
6. Gross, J. *et al.* Dynamic imaging of coherent sources: Studying neural interactions in the human brain. *Proc. Natl. Acad. Sci. U. S. A.* **98**, 694–9 (2001).
7. Nolte, G. The magnetic lead field theorem in the quasi-static approximation and its use for magnetoencephalography forward calculation in realistic volume conductors. *Phys. Med. Biol.* **48**, 3637–52 (2003).
8. Brovelli, A. *et al.* Beta oscillations in a large-scale sensorimotor cortical network: directional influences revealed by Granger causality. *Proc. Natl. Acad. Sci. U. S. A.* **101**, 9849–54 (2004).
9. Dhamala, M., Rangarajan, G. & Ding, M. Analyzing information flow in brain networks with nonparametric Granger causality. *Neuroimage* **41**, 354–62 (2008).
10. Haufe, S., Nikulin, V. V, Müller, K.-R. & Nolte, G. A critical assessment of connectivity measures for EEG data: a simulation study. *Neuroimage* **64**, 120–33 (2013).
11. Oswal, A. *et al.* Deep brain stimulation modulates synchrony within spatially and spectrally distinct resting state networks in Parkinson's disease. *Brain* **139**, 1482–1496 (2016).
12. Akram, H. *et al.* Subthalamic deep brain stimulation sweet spots and hyperdirect cortical connectivity in Parkinson's disease. *Neuroimage* **158**, 332–345 (2017).
13. Smith, S. M. *et al.* Advances in functional and structural MR image analysis and implementation as FSL. in *NeuroImage* **23**, S208–S219 (Academic Press, 2004).
14. Andersson, J. L. R., Skare, S. & Ashburner, J. How to correct susceptibility distortions in spin-echo echo-planar images: Application to diffusion tensor imaging. *Neuroimage* **20**, 870–888 (2003).
15. Andersson, J. L. R. & Sotiropoulos, S. N. An integrated approach to correction for off-resonance effects and subject movement in diffusion MR imaging. *Neuroimage* **125**, 1063–1078 (2016).
16. Dayan, P. & Abbott, L. *Theoretical neuroscience: computational and mathematical modeling of neural systems*. (MIT Press, 2001).
17. Moran, R. J. *et al.* A neural mass model of spectral responses in electrophysiology.

Neuroimage **37**, 706–720 (2007).

18. Nambu, A. Somatotopic Organization of the Primate Basal Ganglia. *Front. Neuroanat.* **5**, (2011).
19. Johnson, M. D. & McIntyre, C. C. Quantifying the neural elements activated and inhibited by globus pallidus deep brain stimulation. *J. Neurophysiol.* **100**, 2549–2563 (2008).
20. Buckwar, E. Introduction to the numerical analysis of stochastic delay differential equations. *J. Comput. Appl. Math.* **125**, 297–307 (2000).
21. Higham, D. J. *An Algorithmic Introduction to Numerical Simulation of Stochastic Differential Equations*. *Society for Industrial and Applied Mathematics* **43**, (2001).
22. Pavlides, A., Hogan, S. J. & Bogacz, R. Computational Models Describing Possible Mechanisms for Generation of Excessive Beta Oscillations in Parkinson's Disease. *PLoS Comput. Biol.* **11**, e1004609 (2015).
23. Litvak, V. *et al.* Resting oscillatory cortico-subthalamic connectivity in patients with Parkinson's disease. *Brain* **134**, 359–74 (2011).
24. Fogelson, N. *et al.* Different functional loops between cerebral cortex and the subthalmic area in Parkinson's disease. *Cereb. cortex* **16**, 64–75 (2006).
25. Holgado, A. J. N., Terry, J. R. & Bogacz, R. Conditions for the generation of beta oscillations in the subthalamic nucleus-globus pallidus network. *J. Neurosci.* **30**, 12340–52 (2010).
26. Tinkhauser, G. *et al.* The modulatory effect of adaptive deep brain stimulation on beta bursts in Parkinson's disease. *Brain* **140**, 1053–1067 (2017).
27. van Ede, F., Quinn, A. J., Woolrich, M. W. & Nobre, A. C. Neural Oscillations: Sustained Rhythms or Transient Burst-Events? *Trends in Neurosciences* **41**, 415–417 (2018).
28. Baker, A. P. *et al.* Fast transient networks in spontaneous human brain activity. *Elife* **2014**, (2014).
29. Goetz, C. G. *et al.* Movement Disorder Society-sponsored revision of the Unified Parkinson's Disease Rating Scale (MDS-UPDRS): scale presentation and clinimetric testing results. *Mov. Disord.* **23**, 2129–70 (2008).
30. Folstein, M. F., Folstein, S. E. & McHugh, P. R. 'Mini-mental state'. A practical method for grading the cognitive state of patients for the clinician. *J. Psychiatr. Res.* **12**, 189–198 (1975).
31. Lambert, C. *et al.* Confirmation of functional zones within the human subthalamic nucleus: patterns of connectivity and sub-parcellation using diffusion weighted imaging. *Neuroimage* **60**, 83–94 (2012).
32. Shink, E. & Smith, Y. Differential synaptic innervation of neurons in the internal and external segments of the globus pallidus by the GABA- and glutamate-containing terminals in the squirrel monkey. *J. Comp. Neurol.* **358**, 119–141 (1995).
33. Nambu, A. Globus pallidus internal segment. *Progress in Brain Research* **160**, 135–150 (2007).
34. Parent, M. & Parent, A. The pallidofugal motor fiber system in primates. *Park. Relat. Disord.* **10**, 203–211 (2004).
35. DeLong, M. R. Primate models of movement disorders of basal ganglia origin. *Trends*

Neurosci. **13**, 281–5 (1990).

36. DeLong, M. R. & Wichmann, T. Circuits and circuit disorders of the basal ganglia. *Archives of Neurology* **64**, 20–24 (2007).

37. Wichmann, T. & Delong, M. R. Functional and pathophysiological models of the basal ganglia. *Curr. Opin. Neurobiol.* **6**, 751–758 (1996).

38. Kita, H., Tachibana, Y., Nambu, A. & Chiken, S. Balance of monosynaptic excitatory and disynaptic inhibitory responses of the globus pallidus induced after stimulation of the subthalamic nucleus in the monkey. *J. Neurosci.* **25**, 8611–8619 (2005).

39. Devergnas, A. & Wichmann, T. Cortical potentials evoked by deep brain stimulation in the subthalamic area. *Front. Syst. Neurosci.* **5**, 30 (2011).
